# Zur: Zinc-Sensing Transcriptional Regulator in a Diverse Set of Bacterial Species

**DOI:** 10.3390/pathogens10030344

**Published:** 2021-03-15

**Authors:** Divya Kandari, Hemant Joshi, Rakesh Bhatnagar

**Affiliations:** 1 Molecular Biology and Genetic Engineering Laboratory, School of Biotechnology, Jawaharlal Nehru University, New Delhi 110067, India; divya43_sbt@jnu.ac.in (D.K.); hemant17_sbt@jnu.ac.in (H.J.); 2 Banaras Hindu University, Banaras 221005, India

**Keywords:** Zinc (Zn), Zn homeostasis, Zinc uptake regulator (Zur), Zur regulon, transcriptional factor

## Abstract

Zinc (Zn) is the quintessential d block metal, needed for survival in all living organisms. While Zn is an essential element, its excess is deleterious, therefore, maintenance of its intracellular concentrations is needed for survival. The living organisms, during the course of evolution, developed proteins that can track the limitation or excess of necessary metal ions, thus providing survival benefits under variable environmental conditions. Zinc uptake regulator (Zur) is a regulatory transcriptional factor of the FUR superfamily of proteins, abundant among the bacterial species and known for its intracellular Zn sensing ability. In this study, we highlight the roles played by Zur in maintaining the Zn levels in various bacterial species as well as the fact that in recent years Zur has emerged not only as a Zn homeostatic regulator but also as a protein involved directly or indirectly in virulence of some pathogens. This functional aspect of Zur could be exploited in the ventures for the identification of newer antimicrobial targets. Despite extensive research on Zur, the insights into its overall regulon and its moonlighting functions in various pathogens yet remain to be explored. Here in this review, we aim to summarise the disparate functional aspects of Zur proteins present in various bacterial species.

## 1. Zinc: An Indispensable Transition Metal

Zinc (Zn) metal is both vital and pernicious for all kingdoms of life. It is a redox stable d-block metal, which possesses a filled d orbital, or a stable oxidation state, eliminating the probability of redox reactions as in copper and iron [1]. A multitude of processes inclusive of critical ones for cellular survival and proliferation, such as DNA synthesis, DNA damage repair, cell division, fighting oxidative stress, and signalling during infection, all involve Zn. It serves as an electrophile or Lewis acid in most of the hydrolytic reactions, thereby it catalyses them, and is also incorporated into a variety of metalloenzymes and storage proteins [2,3,4]. In addition to that, it binds to certain proteins which play a key role in gene expression such as ribosomal proteins, RNA polymerases, tRNA synthetases, certain specific transcription factors and sigma factor interacting proteins [3]. It averts the free radical formation and works as an antioxidant protecting the sulfhydryl groups from the free radical attack. Apart from various catalytic and regulatory roles that it performs, it acts as a scaffold for several proteins like regulatory proteins and transcription factors containing Zn-binding domains like Zn finger, RING and LIM domains [5]. The proteome of organisms living at higher temperatures is rich in Zn-binding proteins, this is probably due to increased utilization of this metal for structural stability of the proteins [6]. 

Zn dependency varies from 5–6% for bacterial proteins to 9–10% for eukaryotic proteins [6,7]. Additionally, 10% of the human proteome is known to bind Zn. Interestingly, the entire set of Zn-binding proteins in an organism is found to be proportional to the total proteome of that organism [6]. The total amount of Zn dispersed in the human body is nearly 2–4 grams [8]. For humans, Zn is additionally needed for immune response modulation, fetal growth, in addition to other cardinal processes of cellular survival. In bacteria, the metal concentration is averagely around 0.1 to 1 mM [4,9]. Considering the crucial roles served by Zn, it is speculated that under Zn famine conditions, many key cellular and metabolic processes could be hampered. Contrarily, despite the pivotal roles that Zn plays in the general cellular metabolism, its excess beyond the physiological threshold of the organism can prove extremely pernicious. Zn in excess promotes the formation of hydroxyl radicals which in turn deteriorates DNA, lipids, proteins, and could also inhibit the aerobic respiratory chain [10,11,12]. Additionally, it has a strong ability to bind proteins in comparison to other divalent metals, hence, its excess intracellular concentrations can give rise to pleiotropic effects in the cellular proteome due to mismetallation, causing alterations in the expression or activity of Zn containing proteins [13]. Overall, the varied roles played by Zn within the organism highlight the importance of its homeostasis for survival. 

### 1.1. Zn and Bacterial Pathogenesis

In a few bacterial pathogens, in addition to supporting the basic survival machinery, Zn is also involved in the execution of pathogenesis ability. For instance, studies have found Zn to promote the biofilm formation in *Pseudomonas aeruginosa* and *Xylella fastidiosa;* additionally, in *Streptomyces coelicolor,* the levels of Zn were found to affect the production of antibiotics and sporulation, all these are indicative of the role Zn plays in their pathogenesis [14,15,16,17]. It is well known that Zn is capable of forming complexes with certain antibiotics, leading to their degradation or inactivation, like with penicillin and tetracyclines [18,19]. Furthermore, metalloproteases possess a broad proteolytic ability and they facilitate the disruption of the physiological barriers during the host invasion by pathogen, thereby assisting in the pathogenesis. Interestingly, the majority of these enzymes are virulence factors as well as being Zn-dependent [7]. Thus, Zn is known to play key roles in bacterial pathogens.

### 1.2. Maintenance of Zn Homeostasis Is Crucial

As there are a plethora of diverse roles played by Zn, it is imperative to uphold the intracellular “free” Zn levels, which accentuates the need for appropriate cellular machinery dedicated towards the maintenance of its homeostasis within organisms. Therefore, both vertebrate hosts and bacterial pathogens have evolved dedicated processes for the sustenance of Zn homeostasis. Understanding the mechanisms employed by the bacteria, specifically the pathogenic ones, might aid in devising newer strategies to tackle them.

Here in this review, we would briefly discuss the importance of Zn in the interaction of vertebrate hosts and pathogens, including understanding the role of Zn in mounting immune responses in host along with the ability of the pathogen to maintain Zn homeostasis to ensure its survival. 

Zn homeostasis in bacteria is achieved through mechanisms like intracellular Zn buffering and intracellular Zn sensing by Zn-responsive transcription factors. These Zn-sensing transcriptional factors are either involved in Zn uptake or its export/intracellular sequestration. While uptake of Zn is regulated by proteins such as zinc uptake regulator, also known as Zur (Fur family transcriptional regulator) or AdcR (MarR/SlyA family), the export/sequestration is regulated by proteins like ZntR or members of ArsR/SmtB families [20]. These transcriptional factors sense the intracellular Zn concentrations by binding to available Zn, which can then either enhance or decrease their DNA binding ability hence altering the transcription of the genes. Additionally, we would discuss the basic structure of this protein and its mechanism of operation in various bacteria, inclusive of the factors that regulate its expression. Further, we would understand the genes that are part of the Zur regulon in bacteria and also those that are indirectly under Zur regulation. We would further explore the role played by ‘Zur’ and the ‘genes regulated by it’ in virulence of certain pathogenic bacteria and conclude with discussing the future possibility of Zur as an antimicrobial target, being crucial for Zn homeostasis. 

## 2. Role of Zn in the Host-Pathogen Interaction

### 2.1. Role of Zn in the Host 

Zn gained appreciation due to the immense potential it holds for all life forms, for example, in humans for fighting various infections and in pathogenic bacteria for survival under harsh host environments. In higher eukaryotes, Zn is involved in diverse aspects of the immune system and its optimal functioning. Interestingly, collagenases and gelatinases, belonging to the matrix metalloproteinases (MMPs) class of enzymes, are all Zn-binding proteins. These enzymes assist in the auto-elimination of dead and damaged tissue and keratinocyte migration in the wound healing process. Zn-binding cysteine-rich metallothioneins possess anti-oxidant activity, which generally confers resistance against the bacterial toxins, as well as reactive oxygen species in human hosts [21].

Additionally, Zn has the potential to regulate acute inflammatory responses, as well as immune and gastrointestinal functions in humans. An appropriate amount of Zn supplemented to burn victims was found to dramatically enhance wound healing, as well as their ability to evade the infections, thereby decreasing mortality [22]. Recently, a study highlighted the importance of dietary Zn in the control of ulcerative colitis disease, wherein Zn supplementation led to a positive clinical outcome [23]. Overall, there is a plethora of evidence demonstrating the dire requirement of this metal in the survival processes, including wound healing, mounting of immune responses and anti-infective action in humans [21,24]. 

Nutritional immunity is one of the host defensive strategies to limit the survival of the invading organism [7,25]. Under the condition of pathogen infection, nutritional immunity involves either abstaining or intoxicating the pathogen of critical nutrients. Usually, metal ions like Fe and Zn are sequestered and made inaccessible to the bacteria [26]. 

### 2.2. A Host–Pathogen Tug of War for Zn

Zn is imperative for both the host and pathogen, thus, the pathogen attempts to access the host Zn reservoirs and in counteraction the host employs defences by either limiting the Zn availability or intoxicating the pathogen with Zn excess, as it is a fact that Zn in higher concentrations is toxic to the cells [27,28,29]. Numerous eukaryotic Zn-binding proteins have been found to possess both Zn^2+^ chelating and pro-inflammatory properties, thereby assisting in the host-mediated antimicrobial activity; the examples include psoriasin, calprotectin and calgranulin C [29,30,31,32]. Calprotectin possesses antimicrobial activity against several bacteria, for instance, *Staphylococcus aureus*, *Candida albicans and Listeria monocytogenes* [30,33,34], which is due to its ability to chelate Zn with a sub-picomolar (pM) affinity [35]. However, pathogens are equipped with mechanisms to counter the host-employed responses at the host–pathogen interface. A study revealed that high-affinity Zn uptake system (ZnuABC) renders survival advantage to *Salmonella enterica serovar* Typhimurium such that the calprotectin-induced Zn scarcity triggers the expression of the *znuABC* operon in the pathogen [36]. *P. aeruginosa* uses the high-affinity ZnuABC Zn transporter system to import Zn ions in response to the host-mediated nutritional immunity [37]. Interestingly, *znuABC* mutant strains of *S.* Typhimurium, *Pasteurella multocida* and *Brucella abortus* were found to be attenuated [38,39,40,41]. This reveals that it is requisite to acquire Zn, using transporters like ZnuABC, for pathogens to survive under Zn limitations due to the host-mediated nutritional immunity. Yersiniabactin and ZnuABC both systems contribute to the Zn acquisition in *Yersinia pestis*. Yersiniabactin is a siderophore which also acquires Zn and expresses under Zn-limiting conditions [42]. There are indeed only a few known Zn uptake mechanisms characterised till date, and ZnuABC is one of them. Numerous other strategies employed by the pathogens to gain competitive advantages over the host microbiome include alternative or additional Zn capture mechanisms that work along with the Zn transporters. ZinT is a soluble Zn trafficking protein, present in some bacterial species like *Escherichia coli*, *Bacillus subtilis* and *Salmonella enterica* Typhimurium. ZinT facilitates the Zn acquisition, as it is capable of delivering Zn ions to the soluble periplasmic domain, ZnuA, of the ZnuABC transporter [26,43,44,45,46]. Another Zn uptake protein called as ZupT is a low-affinity Zn transporter and also member of the ZIP family of proteins. This protein is found in pathogens like *Salmonella enterica* and *E. coli* UPEC, wherein it also serves as a key protein involved in pathogenicity [47,48,49]. There are also certain inner membrane (IM) transporters, namely, ZevAB and ZurAM, implicated in Zn^2+^acquisition in *Haemophilus influenzae* and *Listeria monocytogenes*, respectively [50,51]. 

Additionally, Zn transporters are not solely responsible for maintaining Zn levels in bacteria, besides these influx and efflux transporters, some Zn-binding proteins also capture or acquire Zn. Certain siderophores and metallophores implicated in the acquisition of Zn are, for instance, coelibactin produced by *S. coelicolor* [52] and staphylopine, a small metal-binding metallophore in *S. aureus* [35]. There are also secretion systems in bacteria that are involved in acquiring Zn by secretion of Zn scavengers for the capture of Zn [53]. Some pathogens produce Zn-chelating proteins, which withhold the available Zn ions in the vicinity and transport them to the bacteria. Few pathogens are even smarter to acquire the metal directly from the host molecules harboring the metal, such as, hemoglobin is utilised as the iron source by *S. aureus* [54] and similarly, an outer membrane protein of *Nesseria meningitidis*, namely, CbpA (calprotectin-binding protein A), can capture the human calprotectin and acquire Zn from it [55]. 

Zn is needed for the mounting of an immune response against the pathogen, in addition to imposing toxicity or starvation of Zn on the invading pathogen. Therefore, efficient acquisition and heightened tolerance of Zn contribute towards the virulence of some pathogenic bacteria to ensure survival within its host [56]. Thus, there is a continuous tug of war between the host and the pathogen in a particular microenvironment, and the pathogen tends to adopt various measures to survive the wrath of the host immunity. 

Currently, researchers are exploring the possibilities of targeting Zn homeostasis of bacterial pathogens, the reason being the rapid resistance emergence against the established standard treatment procedures. However, it is a fact that therapeutics directly targeting Zn can severely harm the host along with the pathogen, therefore, understanding the Zn homeostasis and mechanisms in both bacterial pathogens and humans is crucial [57], for devising the pathogen-specific strategies, which is to be discussed in the next section.

## 3. Zn Homeostasis Commences at the Transcriptional Level

It is understood that Zn plays key roles for survival, thus its homeostasis within the organism is imperative. This is primarily achieved through the Zn-reliant changes in the expression of the requisite genes, brought about by Zn-responsive transcriptional factors. These factors are responsible for the requisite reactions to counter Zn fluctuations, achieved through transcriptional regulations on the genes required for Zn storage and transport. Regulating the expression of such genes ensures that the Zn levels are adjusted in tune with the cellular requirements.

**Figure 1:**
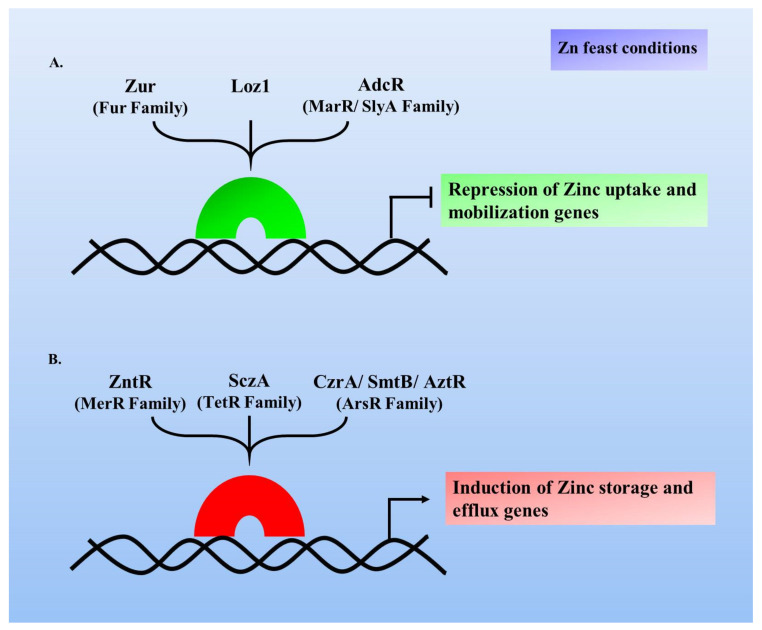
Zn-dependent transcription regulators exert a repressor or an activator function in presence of Zn to achieve Zn homeostasis. (**A**) Zur, Loz1 and AdcR are the transcriptional repressors that mediate Zn-dependent repression and (**B**) ZntR, SczA and CzrA are the transcriptional activators that mediate Zn-dependent activation, under Zn feast conditions.

Metal-sensing transcription factors are present in all life forms; these include several bacterial factors like Zur, ZntR, AdcR and various other eukaryotic factors, including Loz1, MTF-1 and bZip19 [58,59,60]. These factors can be categorised into those either governing Zn uptake to offset Zn deficiency, or those regulating Zn storage or its efflux to prevent cellular toxicity with its excess. Transcriptional repressors like Zur, Loz1 and AdcR are involved in Zn-dependent repression of Zn uptake genes (Figure 1A). Explicitly, under Zn scarcity, these factors lose their bound Zn and undergo conformational changes, thus losing their binding ability to Zur box, thereby causing the target gene derepression and when Zn repletes, these factors regain their active ‘Zur box binding’ form by binding Zn, leading to repression of the target genes [61,62]. On the contrary, SczA, ZntR, SmtB being the Zn-dependent activators protect from Zn-excess by triggering the expression of the Zn efflux or storage proteins (Figure 1B). For example in case of *B. subtilis*, CzrA protein (an ArsR/SmtB family member) is a transcriptional activator, whose regulon expresses under Zn excess conditions and similarly, ZntR (a MerR transcriptional regulator family member) of *E. coli* functions to alleviate metal toxicity. Thus, these transcription factors respond to the Zn fluctuations within the cell and manage optimal levels of Zn. This review article describes Zur, a transcriptional factor with disparate aspects in a diverse set of bacterial species, playing crucial roles under varied Zn concentrations, as well as in the other arenas of bacterial life.

## 4. Zinc Uptake Regulator 

### 4.1. Background of Zur

Zur (Zinc Uptake Regulator) is a Zn-sensing transcriptional regulator that is a member of the FUR superfamily of metal-sensing transcriptional regulators. Although its name implies that it regulates Zn import, Zur also maintains the homeostasis of Zn [63], in response to the fluctuations in the intracellular Zn levels. This is achieved by regulating the expression of its target genes. 

FUR superfamily members are ubiquitously present in prokaryotes, such as firmicutes, cyanobacteria and other major phyla. In addition to Zur, the other members of this family are Fur (Ferric Uptake Regulator) [64,65], PerR (Peroxide Regulator) [66,67], Nur (Nickel Uptake Regulator) [68] and Mur (Manganese Uptake Regulator) [69,70] proteins. Zur was initially identified in *E. coli* [71] as a pivotal protein for Zn homeostasis and further in other organisms including *B. subtilis* [72,73,74], *L. monocytogenes* [75], *Xanthomonas campestris* [76], *S. coelicolor* [17], *Streptococcus sp.* [77], *Enterococcus faecalis* [78] and *Mycobacterium tuberculosis* [79].

### 4.2. Zur and the Intracellular Zn

**Figure 2:**
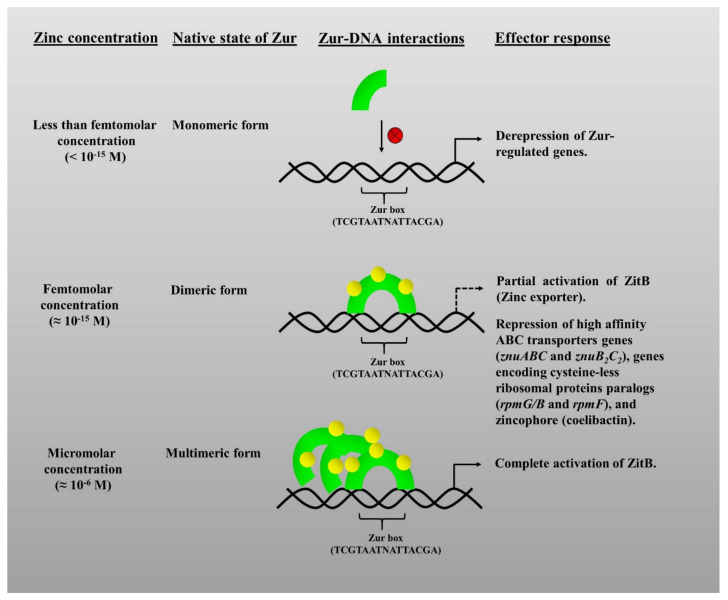
Schematic representation of the graded response of Zur under varying Zn concentrations [62]. The yellow spheres represent Zn metal. Zur loses its DNA-binding affinity at Zur box and causes derepression of its regulon genes when Zn is “less than femtomolar concentrations”. Under femtomolar concentrations of Zn, Zur binds to the DNA and partially activates Zn exporter gene (*zitB*) and represses the genes for high affinity ABC transporter like *znuABC* and *znuB_2_C_2_* and cysteine-rich ribosomal proteins like rpmG/B and rpmF. Under micromolar concentrations, Zur completely activates *zitB*.

Zur, being a transcriptional regulator, regulates its regulon genes in response to the reversible binding of Zn. Zur family proteins are sensitive to intracellular Zn concentrations, with its Zn-binding sites having a femtomolar (fM) affinity (10^−15^) for Zn [4]. The affinity of Zur proteins for Zn mistakenly led to the idea that free intracellular Zn concentrations lie in the femtomolar range, whereas the free intracellular Zn concentrations are much higher, that is, in the picomolar range (10^−12^), as found in *E. coli* [80]. “Free Zn” is a term referring to the “rapidly exchangeable Zn” available in the cell, which could be bound to weak ligands. However, the overall Zn concentrations are much higher, that is in the millimolar range (10^−3^). The variation between the cellular Zn concentrations and the sensitivity of Zur does create an aberration in understanding, however, it was reasoned with explanations in previous reports. In *B. subtilis*, the increase in concentrations of Zn, like from femto– to picomolar and pico– to micromolar (µM), causes its subsequent binding to different Zn-binding sites in Zur. This was correlated to the graded response of Zur in the expression of various genes [81]. In *S. coelicolor*, under femtomolar Zn concentrations, Zn binds to the three high-affinity Zn-binding sites in Zur to allow it to exert repression over its target genes. However, beyond its affinity range, the Zur successively forms oligomers and multimers as the concentrations rise from femto-to pico- and further to micromolar (Figure 2) [62]. Therefore, it undoubtedly points towards the extreme sensitivity and high Zn-binding affinity of Zur [81].

### 4.3. Zur in Bacterial Species 

**Figure 3:**
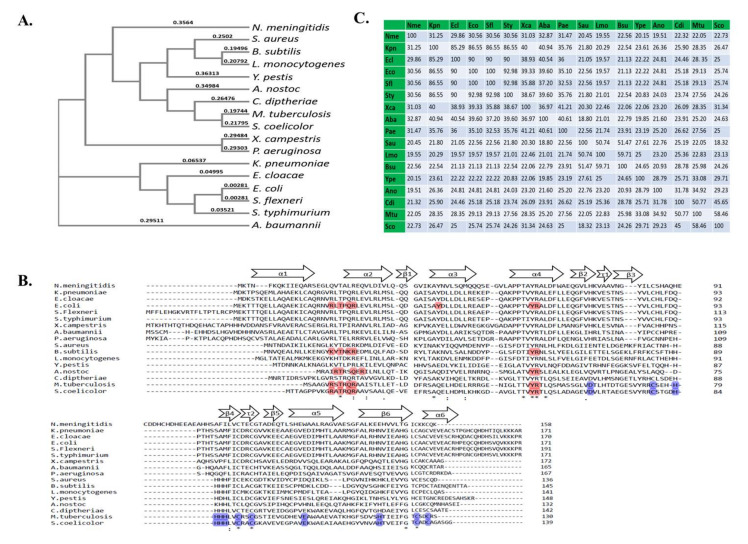
Sequence analysis of the Zinc uptake regulator (Zur). (**A**) Phylogenetic tree of Zur proteins. The tree was constructed based on multiple sequence alignment of Zur proteins from different organisms, using CLUSTAL O. (**B**) Amino acid identity matrix of Zur proteins from *Neisseria meningitides (Nme)*, *Staphylococcus aureus (Sau)*, *Bacillus subtillis (Bsu)*, *Listeria monocytogenes (Lmo)*, *Yersinia pestis (Ype)*, *Anabaena nostoc (Ano)*, *Cornybacterium diptheriae (Cdi)*, *Mycobacterium tuberculosis (Mtu)*, *Streptomyces coelicolor (Sco)*, *Xanthomonas campestris (Xca)*, *Pseudomonas aeruginosa (Pae)*, *Klebisella pneumonia (Kpn)*, *Enterobacter cloacae (Ecl)*, *Escherichia coli (Eco)*, *Shigella flexneri (Sfl)*, *Salmonella* Typhimurium *(Sty)* and *Acinetobacter baumanii (Aba)* created through CLUSTAL 12.1 program. (**C**) Sequence alignment of Zur proteins. The sequence of Zur proteins from different species was aligned. Red highlighted residues are involved in DNA binding confirmed through X-ray crystallography. Thr-13, Tyr-52 and Arg-53 of ScoZur are conserved and Arg-16 was found to be semi-conserved in other species. Residues of the metal binding site are highlighted in blue. The metal binding site constituted by Cys-90, Cys-93 Cys-130 and Cys 133 of ScoZur are conserved in other bacterial species as well ( Shin et al. 2011).

We compared the Zur proteins from different bacterial species, as shown in Figure 3 by phylogenetic tree (a), identity matrix (b) and multiple sequence alignment (c). These are inclusive of those bacterial species in which Zur is not characterised till date, namely, *Shigella flexneri, Klebsiella pneumoniae* and *Enterobacter cloacae*. These pathogens are all capable of causing various infections in humans, as we know, *S. flexneri* causes diarrhoea, *K. pneumoniae* causes pneumonia and *E. cloacae* causes a range of infections like urinary tract infections, osteomyelitis and ophthalmic infections. The high level of identity of these proteins with the Zur of human pathogen *S. enterica* Typhimurium, wherein Zur is needed to survival within host, is interesting [82,83]. 

Bacteria being single-celled entities are separated from their immediate environment by a set of cellular membranes, so it is of utmost importance to maintain the optimal intracellular Zn levels despite the extracellular milieu conditions. The mechanisms for the channelling of Zn ions include its import, sequestration, intracellular trafficking by metallochaperones and selective export across the cytoplasmic membrane. The extent of influx and efflux channels present to control the fluxes of the metal, which are majorly controlled at the transcriptional level by Zur. 

### 4.4. Zur-Mediated Zn Homeostasis 

**Figure 4:**
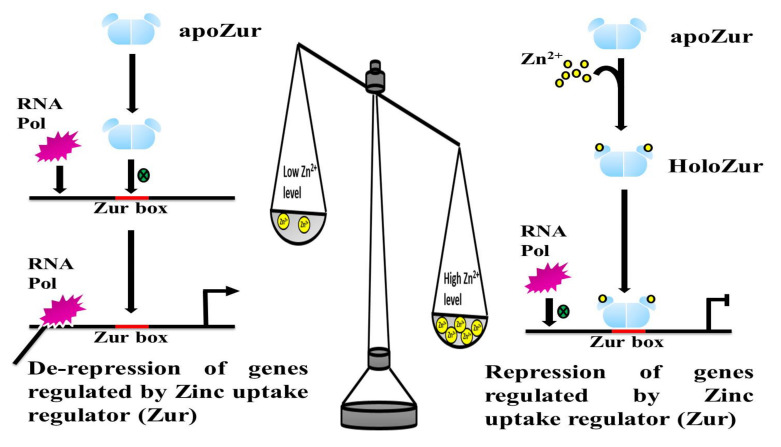
Schematic representing the mechanism of Zur under Zn-limiting and Zn-excess conditions. Under Zn excess conditions, binding of Zn ions (represented by yellow color spheres) to Zur dimer (blue) causing transcriptional repression and under Zn limiting conditions, the repression is relieved.

Zn-binding alters the conformation of Zur, which then enhances its affinity to bind the cognate DNA. Zur is a transcriptional factor that senses the Zn levels and accordingly binds or unbinds its cognate DNA, causing relevant transcriptomic alterations. Previously, structural and mutagenesis analysis of Zur proteins suggested that it is present as an inactive dimer that binds to one structural Zn (II) ion per monomer [81,84]. Under Zn replete conditions, Zur binds to one or more regulatory Zn, depending on the organism, to form a fully active repressor with a high affinity for its cognate DNA. Hence, the Zur proteins “sense” the intracellular Zn concentrations, thereby leading to simultaneous alterations in its DNA-binding affinity. Therefore, under Zn sufficiency in the cell, Zur molecules bind to it and undergo a conformational change that augments its DNA binding ability, hence keeping the genes involved in Zn uptake and mobilization in a repressed state (Figure 4). However, Zur-mediated repression is released when the cell encounters a Zn-depleted condition, thereby initiating Zn uptake and mobilization [71]. Hence, maintenance of Zn homeostasis by Zur is not merely through controlling the expression of the metal uptake systems, rather, it is a collective process of regulating genes encoding for Zn transporters (both low—and high-affinity), chaperone proteins, non-Zn-binding paralogs of ribosomal proteins, and various other proteins involved in Zn homeostasis [63].

## 5. Structure and the Molecular Mechanisms of Zur 

### 5.1. Structural Aspects of Zur

X-ray crystallographic structures of Zur proteins from bacteria like *S. coelicolor* (ScZur), *E. coli* (EcZur) and *M. tuberculosis* (MtZur) [81,84,85] have been elucidated. These structural studies revealed the presence of an N-terminal DNA binding (DB) domain and a C-terminal dimerization (D) domain, which pair up with another monomer to generate a “dimer”. An interdomain hinge region is present which usually houses a metal-binding site referred to as M-site. The other metal-binding sites could be located in the surface of D domain called D-site and in proximity to the C-terminus, therefore, called the C-site [81]. Zur binding to DNA is again assumed to be through cooperative binding among the two Zur monomers as in the case of EcZur [85].

Dimeric Zur, as in the elucidated structures, could adopt two conformations depending upon the availability of Zn. The “closed” conformation is with bound Zn and is reported to have the high binding affinity, whereas “open” lacks bound Zn and is reported to have low binding affinity at the consensus site of cognate DNA [20]. EcZur has two Zn-binding sites, whereas MtZur and ScZur each bear three sites per monomer; however, not all the sites are involved in Zn sensing. According to the structural studies conducted on ScZur, there are two regulatory sites and one structural site in this protein. The C-site constituted by four Cys residues was referred to as the structural Zn-binding site and the M- and D-sites as the regulatory sites [81]. Further, through biochemical analysis, it was revealed that the C-site is crucial for the structural integrity and mutations in this Zn-binding site led to alterations in the secondary structure and loss of dimerization ability of the protein. Various other studies have supported this observation, wherein Zn performs a structural role and the site similar to the C-site, as above, is responsible for maintaining the protein dimer [86,87,88,89]. The M- and D-sites are involved in regulatory functions. In fact, Zn-binding to the M-site was found crucial for Zur activity such that the absence of Zn at the M-site rendered it unable to bind to its target genes. 

### 5.2. The Graded Response of Zur

Interestingly, it was revealed that binding of Zn to the M-site, as discussed above, alone is not enough for the full-fledged function of Zur. The presence of the third metal-binding site (D site) in bacterial Zur proteins imparts to them the ability to possess a three-staged differential activity to achieve a fine-tuned response against different extents of Zn starvation. It is referred to as “graded response” [74] wherein the number of Zn ions bound to the available Zn sites in the protein affects its DNA binding ability. Interestingly, differential effects on the expression of target genes could be observed on mutational analysis of the D-site. It was identified that binding of Zur to certain promoters referred to as sensitive promoters (in case of ScZur they are znuA and rpmF2 promoter sequences) required higher Zn levels. So, when cellular Zn concentrations were ample, then binding of Zn to the D-site of Zur caused the repression of these sensitive promoters and a slight decrease in Zn led to the derepression of these promoters and thereby expression. Zur as a metal-responsive transcriptional regulator, effectively fine-tunes the Zn concentrations within the cell by graded expression of the Zn uptake and mobilization genes, in accordance with the cellular availability of Zn [81]. Hence, as the cells undergo a transition from the Zn suffices to Zn limitation that the Zur derepresses its regulon genes in a graded manner, leading to the sequential expression of the appropriate genes depending on the degree of Zn deficiency, rather than an immediate step (Figure 2). Mobilizing Zn is found to be the foremost resort for the cell under initial Zn limiting conditions and further options include uptake mechanisms that are triggered under continued limiting conditions, as observed in *B. subtilis* [74] and *S.* Typhimurium [90]. Further investigation is still needed to get insights into the exact mechanisms behind the graded response of Zur during the transition from sufficiency to the limitation of Zn and from limitations to Zn sufficiency.

### 5.3. Molecular Mechanisms of Zur Proteins

While Zn-binding sites in Zur have been well studied in many bacterial species, the residues of Zur involved directly in binding the cognate DNA have not been explored in almost all of them. Recently, the residues of Zur protein of *B. anthracis*, directly involved in the interaction with the consensus Zur box in the promoter of its regulon genes, were identified [91]. Similar studies could be useful for devising strategies to target the DNA binding activity of Zur. In previous studies, the molecular mechanisms of Zur performing the repression of the target genes under Zn-excess and derepression under its scarcity (Figure 4), has been studied in detail. For instance, in *E. coli,* promoters of the target genes have a 30-bp AT-rich sequence called the Zur box, overlapping with its −35 and −10 regions and Zn-bound-Zur protein binds and blocks the entry of RNA polymerase hence suppressing the transcription [71]. Similar mechanisms of action by Zur are in *B. anthracis, B. subtilis, M. tuberculosis* and *S. coelicolor* [17,72,74,79,92] for repression of Zn uptake systems. 

A recent breakthrough study in *E. coli* reports that the unbinding of DNA by Zur is sensitive to the concentration of the Zur protein itself, and this is a first-of-its-kind reaction, which is initially impeded and later facilitated with increasing “non-Zn-bound Zur” concentration. This is contrary to the conventional belief that protein unbinding is independent of free protein concentration and is a unimolecular process. This study also reveals that *E. coli* Zur protein, in its non-Zn form, binds to the chromosome tightly at a non-consensus DNA site [93]. Similar investigations must be carried in other bacterial species, as it would be interesting to get deeper insights into the mechanism of repression and derepression, to compare them between pathogenic and non-pathogenic bacteria. While repression is still explored in detail, little is known about the facilitation of derepression.

Evidently, Zur usually acts as a negative transcriptional regulator of the genes encoding for the Zn uptake systems. However, in a few cases, Zur proteins can also act as activators of transcription as there are instances when the allosteric switch has the opposite effect, that is, increased DNA affinity in the absence of Zn. Zur protein of *X. campestris*, under Zn feast conditions, acts as a transcriptional activator by causing the expression of metal efflux proteins apart from its usual repressor behaviour on the other target genes, under Zn excess. It is probably due to the recognition of a different binding sequence in the promoter region of the genes to activated than those repressed [94]. It is, therefore, speculated that many such Zur-activated genes are present, but they remain unexplored. Similarly, in *S. coelicolor,* Zur (ScZur) in addition to graded also shows a dual-phase response, as per Zn availability. ScZur exerts complete repression over the Zn uptake genes and partially activates the *zitB* gene (involved in Zn export driven by a transmembrane gradient), as a part of its phase I graded response under femtomolar Zn concentrations. However, when the Zn concentrations increase up to micromolar ranges, then Zur forms oligomers or multimers, thereby binding to the ZitB promoter and fully activating its transcription to bring about Zn export (Figure 2) [62].

In nature, a variety of different mechanisms exist in various organisms to achieve one target, in our context, some of the bacterial species as stated above, have Zur protein that can act both as activator or repressor and in rest, there are different metalloregulators for each process. For instance, in *E. coli,* two separate transcriptional regulators function to maintain the intracellular Zn levels, namely, ZntR and Zur, while one responds to its excess, the other to Zn scarce, respectively. Each regulator senses different intracellular Zn concentrations, while it is 0.2 fM for Zur and it is 1.2 fM for ZntR [4]. It is therefore quite evident that our understanding and insights into the structures, dynamics and biomolecular complexes of Zur is as yet far from comprehensive. 

Zur as a transcription factor exerts control over the expression of its regulon genes, but what governs the expression of Zur is important to know. From the previous studies done on Zur, it is evident that in the majority of bacterial species, Zur auto regulates its expression. Till date, autoregulation of Zur has been found in various bacterial species as a regulatory mechanism, like in *Synechococcus sp.* PCC 7002 [95], *Paracoccus denitrificans* [96], *Agrobacterium tumefaciens* [97,98], *Acinetobacter baumannii* [99], *Pseudomonas protegens* [100], *Pseudomonas aeruginosa* [101,102,103], *Enterococcus faecalis* [78], *Staphylococcus aureus* [104], *Listeria monocytogenes* [105], *Streptomyces coelicolor* [15,17,62], *Bacillus anthracis* [92], *Yersinia pestis* [106], *Haemophilus influenzae* [45] and *Haemophilus ducreyi* [107]. 

## 6. Regulon of Zur 

**Figure 5:**
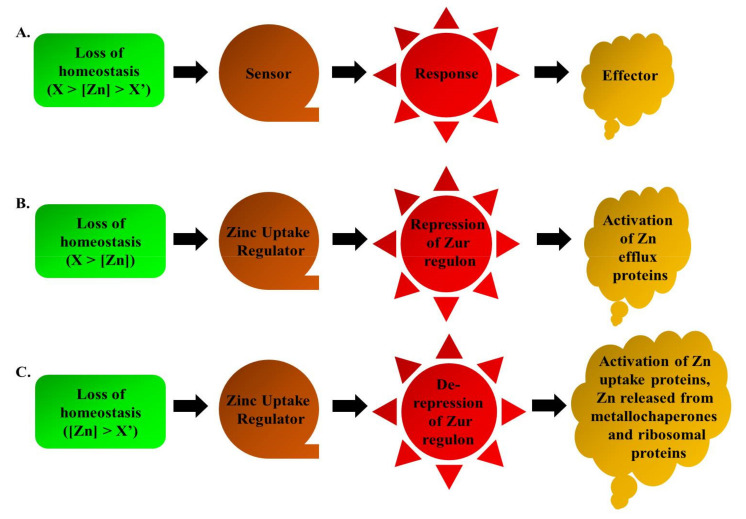
Schematic representation of loss of Zn homeostasis in bacteria. (**A**) Basic outline for loss of homeostasis. Loss of homeostasis (**B**) under Zn excess conditions; (**C**) under Zn limiting conditions, wherein X corresponds to Zn concentrations greater than the threshold concentration, X’ corresponds to Zn concentrations less than the threshold concentration.

Zur is predominantly known for its repressor activity as a transcriptional regulator. We have briefly discussed about the various Zn-binding sites that could be present in this protein. Thus, the intracellular concentrations of Zn determine the Zn occupancy into the regulatory Zn-binding sites of Zur. This variable degree of Zn binding onto Zur protein is further responsible for its graded response in terms of gene expression. Therefore, it is presumed that the degree of Zn level fluctuation, whether feast or famine, leads to the counter cellular responses in a graded manner. When the cell loses harmony with the Zn levels, it adapts a few measures for its healthy sustenance. These measures are taken in phases corroborating with the loss of homeostasis, which can be either by limitation or excess of this metal ion, as represented in Figure 5. 

### 6.1. The Non-Zn-Binding Paralogs of Ribosomal Proteins

Next, it is known that the ribosomes are the most abundantly occurring macromolecular entities in the cell; interestingly, the C^+^ forms of the ribosomal proteins bind Zn, therefore, in addition to their role in translation, the ribosomes also serve as a storehouse for Zn in the cell. C^+^ forms are the ones which comprise two pairs of conserved CXXC stretch and are hence capable of binding to Zn while their other ribosomal paralogs lack this motif. Zur regulates these non-Zn-binding ribosomal proteins, which replace the Zn-binding ribosomal proteins under Zn starvation conditions, to promote the mobilization of Zn. L31 and L33 are the two Zn-binding ribosomal proteins having non-Zn-binding paralogs, regulated by Zur. Under the Zn famine conditions, the non-Zn-binding variants express and displace the Zn-bound proteins further mobilizing Zn. Each ribosomal unit contains 6–8 equivalents of Zn [108], and taking into account their number per cell, it is inferred that ribosomes are indeed a storehouse of Zn. 

### 6.2. High-Affinity Zn Uptake Systems

Various Zn uptake and efflux systems are under the regulation of Zur, for instance, high-affinity bacterial Zn uptake systems, the most common ones, are the *znuABC* (ATP-binding cassette), also known as ABC transporters, as discussed previously. These transporters have a cytosolic dimeric ATPase, a membrane-spanning dimeric permease and a monomeric substrate-binding protein (SBP), which is highly selective for its substrate metal ion. These transporters are widely present in bacterial species like *E. coli*, *S. coelicolor*, *B. subtilis*, *L. monocytogenes*, *P. aeruginosa*, *Y. pestis*, *Vibrio cholerae*, *A. baumanii*, *X. campestris*, *N. meningitidis* and are regulated by Zur [20,71,74,105,106,109]. The typical gene arrangement of ABC transporter includes the presence of a gene, each for transmembrane permease and an ATPase adjacent to the SBP that is three genes *znuA*, *znuB*, and *znuC,* such that they are co-transcribed in an operon. However, in *B. anthracis, P. aeruginosa*, *Y. pestis*, *H. influenzae*, *H. ducreyi*, *S. coelicolor,* the arrangement deviates from the paradigm and while *znuA* is present as a single gene, *znuB* and *znuC* forms polycistronic transcripts with the *zur* itself. Therefore, in such gene arrangements, *zur* autoregulates its expression being a part of an operon with *znuB* and *znuC,* that is, it represses its transcription under Zn suffice conditions [45,92,103,106,107].

Certain metallophores produced by the bacteria facilitate Zn acquisition and are under Zur regulation. The best-known metallophores are staphylopine generated by *S. aureus* [35], pseudopaline by *P. aeruginosa* [102,110], yersiniabactin by *Y. pesti* [42,111] and coelibactin, which is identified as the first naturally occurring zincophore in *S. coelicolor* [15]. While pseudopaline, staphylopine and coelibactin have been reported to be Zur-regulated, there are no shreds of evidence for the same in yersiniabactin. *Sco7676*, *Sco7681* and *Sco7682,* are the genes of *S. coelicolor*, involved in coelibactin biosynthesis, were previously found to be regulated by Zur [15,62]. Staphylopine is a broad spectrum metallophore in vitro, but in *S. aureus* it functions as a zincophore-chelating Zn, encoded by cntKLMABCDFE operon, which also encodes for ABC transporter which transports it intracellularly in Zn-bound form. This operon is under the regulation of both Fur family transcriptional regulators, namely, Zur and Fur [112]. Pseudopaline, another Zn metallophore, encoded by *cntOLMI* operon, in *P. aeruginosa* [102] is Zur-regulated. Additionally, genes, namely, *queC* and *queF* are also Zur-regulated, these encode for the cytoplasmic enzymes involved in the quenosine production, which is basically a modified nucleoside and is often present at the first/wobble position of the tRNA anticodon. The expression of these genes increases under low-Zn conditions, as reported in *N. meningitidis* [113].

### 6.3. Non-Zn-Binding Paralogs of Enzymes 

Another Zur regulon member is a paralog of FolE enzyme called as FolE2. These are typically involved in the folate biosynthesis but exist in both Zn-dependent and independent forms. The non-Zur regulated FolE is a Zn^2+^-dependent enzyme. Under Zn scarcity, FolE releases the bound Zn as its non-Zn-binding paralog FolE2 replaces it. Thus, the expression of this non-Zn-binding paralog of this enzyme, FolE2, is under Zur regulation. This phenomenon is in bacteria like *B. subtilis* and *P. aeruginosa* [103,114]. Similarly, DksA is a transcriptional regulator that mediates inactivation of transcription of the ribosomal RNA genes and activates the genes involved in the synthesis as well as transportation of amino acids. DksA2 is the non-Zn-binding paralog of DksA regulated by Zur. DksA2 is expressed under the circumstances of Zn deprivation to replace its Zn-binding paralog DksA, releasing Zn for other essential cellular processes. This paralog also serves as a backup copy of the Zn-dependent DksA protein under the Zn-deficit conditions [115]. 

### 6.4. Metallochaperons 

The metallochaperones have variable specificities for binding to the metal ions and are responsible for the appropriate metal allocation. G3E GTPase superfamily comprises of various metallochaperons and it is divided into subfamilies, of which COG0523 is one [116]. Many of the COG0523 subfamily proteins are Zur-regulated and are implicated in Zn homeostasis. These proteins are well known to possess GTPase activity and can also bind Zn, hence, in addition to their usual functions, they also support the essential metabolic processes by supplying Zn to them under Zn-famine conditions. YciC of *B. subtilis* (ZagA/BsuYciC) is the most reviewed member of the COG0523 subfamily; others are YjiA and YeiR of *E. coli* and ZigA of *A. baumanni.* Two YciC homologs are present in *B. anthracis*, namely, BaYciC and BAS1786, belong to COG0523 family of proteins [45,92,117,118,119,120]. Analogously, in *Pseudomonas putida*, two Zur-regulated COG0523 paralogs are present, of which one belongs to the subfamily 1, and the other to the subfamily 11 [116].

Number of total genes regulated by Zur vary for different bacterial species, while in *C. glutamicum* [121] it is only 9, but for *N. meningitidis* [113] and *M. tuberculosis* [79], it is 17 and 30, respectively. Additionally, in certain cases, the Zur regulon is extremely large, that is, up to 154 and 121 genes present in *Y pestis* and *S. suis* serotype 2 strain [77,122], respectively. 

Predominantly, the usual regulon of Zur in various bacterial species includes genes encoding metal transporters, metal chaperones, Zn-independent ribosomal proteins, and Zn-independent paralogs of some enzymes.

## 7. Zur and Zur-Regulated Genes, Explored for Their Role in Pathogenicity

Delving into the importance of Zn led to finding that deletion of *znuABC* genes in certain bacterial species could dramatically perturb their pathogenicity rather than merely affecting their ability to grow in in vitro environments poor of Zn. These pathogens depend on ZnuABC for infecting their hosts, for instance, *Brucella abortus* [41,123], *A. baumannii* [25], *Campylobacter jejuni* [124], *E. coli* strain [49,125], *H. ducreyi* [107], *Moraxella catarrhalis* [126], *Pasteurella multocida* [40], *S. enterica* [38,39] and *Yersinia ruckeri* [127]. However, in *Y. pestis*, ZnuABC is required for Zn uptake and is essential for growth, but it does not conduce virulence [106]. Presence of other Zn uptake mechanisms that compensate the Zn uptake under host-mediated Zn deficit was assumed to be a possible reason for the dependency of the above-listed pathogens on ZnuABC transporters. Subsequent studies then found yersiniabactin as the other supportive mechanism as the double mutants lacking both *znuABC* and yersiniabactin (*ybtX*) were extremely attenuated [111]. Overall, ZnuABC transporters are present both in Gram-positive and Gram-negative bacteria and are usually virulence determinants. The possibility of employing *znuABC* mutant strains as vaccines, to confer resistance against the wild type infection explored for pathogens like *S.* Typhimurium and *B. abortus,* was successful. Attenuated *S. enterica* serovar Typhimurium strain, lacking the ZnuABC transporter, was found to be a promising vaccine candidate [82]. Various studies corroborated that oral administration with the attenuated *S. enterica* serovar Typhimurium lacking *znuABC*, in both mice and pigs models, imparted to them protective ability against the challenges with the virulent *S.* Typhimurium strain [83,128,129]. Later, the safety and immunogenicity studies of this vaccine were done to prove that it was a promising candidate. Interestingly, strains lacking ZupT and ZinT genes in addition to *znuABC* gene proved to be much safer vaccine candidates [83]. Similarly, ZnuA deletion in *B. abortus* (*B. abortus* ∆znuA) attenuated the virulent strain and through protection studies its potential as a live vaccine was confirmed in mice [41,123]. It would be interesting to explore whether *znuABC* genes are crucial for virulence in other bacterial pathogens as well.

Zur is a multifunctional protein and various evidences support that it regulates expression of a plethora of other genes inclusive of those involved in Zn homeostasis. In various pathogens like *M. tuberculosis, S. coelicolor, Y. pestis, P. aeruginosa, V. cholerae* and *L. monocytogenes,* Zur regulates other kinds of genes which are involved in virulence, than just its conventional regulon, therefore assisting in survival within the host. These additional functions apart from the principal function are called moonlighting functions [130]. Numerous proteins are known to possess this phenomenon and, due to this ability, they are involved in the pathogenesis of the bacteria. For instance, glyceraldehyde-3-phosphate dehydrogenase (GAPD), enolase and Hsp70, all these proteins apart from their usual function play roles in bacterial virulence. Likewise, Zur does play various moonlighting roles in bacterial species. In *B. subtilis*, Zur regulates the expression of the genes which are involved in the transport of amino acids [109]. Zur is indirectly involved in virulence of *S. Typhimurium*, wherein it regulates *fliAZ* gene, which in turn positively regulates the invasion genes [39,131]. Interestingly, disruption of *zur* in *S.* Typhimurium led to a nearly 10-fold increase in the mice LD_50_ (lethal dose, 50%) [131]. Similarly, in a previous study, a *zur*-disrupted mutant of *X. campestris* led to attenuation of its virulence and decreased production of extracellular polysaccharide (EPS) which is key to its virulence [76,132]. The reduction in virulence was not as a result of intolerance to Zn deprivation or toxicity, as may be speculated from the clear roles played by Zur as a Zn-responsive transcriptional regulator. Thus, the *zur* disruption led to altered expression of a few unidentified genes which were involved in/related to its virulence [76]. Screening for virulence-associated genes of *P. aeruginosa* led to the identification of a *zur*-like gene, *np20*, which when disrupted resulted in the reduction of virulence in the mouse models [71]. ZnuABC, in addition to being involved in uptake of Zn, is also associated with virulence in *P. aeruginosa* [133]. In *V. cholerae*, the search for the genes induced during infection resulted in the identification of a gene that held significant similarity to *zur* [134]. Deletion of *zurR* (*zur*-like gene) in *L. monocytogenes* led to significant attenuation of virulence in addition to the influences on cell size, motility and resistance to toxic Zn levels [105]. In *M. tuberculosis*, genes encoding the secretory proteins, such as early secretory antigen target 6 (ESAT-6) and culture filtrate protein (CFP 10), that are involved in virulence, are regulated by Zur [79]. Likewise, Zur regulates the expression of α-hemolysin toxin as a virulence factor encoded by *hlyCABD* operon in uropathogenic *E. coli* [135]. Further, in *S. coelicolor*, Zur regulates a cluster of genes responsible for the synthesis of a siderophore-related peptide called coelibactin, deregulation of which inhibits sporulation [15]. Virulence-related genes like, *rovA*, *psaEF*, *psaA* and *ail,* are under Zur regulation in *Y. pestis* [122]. Thus, numerous studies have highlighted the diverse ways of involvement of *zur* in virulence of many pathogens, but for the rest, it is a matter that merits further investigation.

## 8. Scope of Zur Protein as a Putative Antimicrobial Target 

Zur is identified in many bacterial species till date and studies are uncovering the importance of this protein in various aspects of the life of pathogens. An antimicrobial agent can only be effective in eliminating the pathogen if it targets essential or conditionally essential processes of the pathogen and additionally, the target should be highly specific lacking any possible homolog within the host, thereby lowering the chances of adverse effects on the hosts. Antimicrobials that are effective against a large number of bacterial species are preferred to achieve the broad-spectrum antibacterial activity. The hunt for novel antimicrobials has intensified, given the burgeoning incidences of antimicrobial resistance. When questions arise on the ability of Zur to act as a potential antimicrobial target, there is a lack of any conclusive or direct evidence. 

We know that when the host employs nutritional immunity upon infection, Zur-mediated activation or repression of Zn uptake or efflux proteins insulates the pathogen from the fluctuations in the metal concentrations. Zur performs cardinal roles, highlighting its importance in bacterial systems, but the effectiveness of Zur as a therapeutic target remains majorly uncharted. Previously, a study on *S. enterica* reported the identification of two inhibitor molecules, namely, RDS50 and RDS51, against ZnuA, which were capable of causing growth inhibition in the bacterium. This points towards the crucial status that Zn has and knocking off any of the systems that impair its homeostasis duly affects the growth and pathogenesis [136]. Additionally, it has been discussed previously that the znuABC mutant strain of *S. enterica* is attenuated and hence is a potent vaccine candidate. 

Fur belongs to the same superfamily as of the Zur. Fur plays a significant regulatory role in Fe homeostasis in many bacteria and is absent in eukaryotes. Notably, through various studies, it was found that the *fur* mutants of different pathogenic bacteria like *V. cholera*, *P. aeruginosa* and *S. aureus* demonstrated decreased virulence [137,138,139]. Attenuated virulence because of the absence of Fur probably makes it a potent antimicrobial target. Further, small anti-Fur peptides were designed, which could effectively inhibit Fur as an anti-virulence strategy against pathogens [140]. Studies have found Zur to be directly or indirectly linked to the virulence of some pathogens, as explained in detail in the previous section. All this suggests Zur to be a suitable nominee in the category of antibiotic targets.

In summary, studies throughout the world relating the zinc uptake regulator or any similar regulator governing Zn homeostasis are majorly being conducted with a view to discovering their underlying potential as an antipathogenic target. Interestingly, although nature has made Zn critical for all, the regulatory mechanisms are separate for both eukaryotes and prokaryotes, even though they all function to achieve threshold levels of this metal. Zur protein is widely present in many bacterial species and it has been seen that it has quite high levels of homology among its various orthologs (Figure 3); certainly, however, it has numerous underlying moonlighting functions in different species, which are yet to be discovered. However, the predominant role of Zur is to transcriptionally regulate its regulon genes to maintain Zn homeostasis (Figure 5). Noticeably, it imparts the ability of enhanced virulence in some pathogens, while in others it either has few roles different from its usual function of maintaining Zn homeostasis. Overall, it is an interesting research area, and we assume that in the future this protein could be exploited to develop a suitable targeted therapy against pathogens.

## 9. Conclusions 

Zn has tremendous importance in all life forms. Since it is essential but deleterious in excess, during the course of evolution, the living organisms have devised strategies or regulatory mechanisms to cope up with the fluctuations in concentrations of this metal. Interestingly, the mechanisms of regulation are usually different in prokaryotes and eukaryotes. Apparently, such bacterium-specific mechanisms can be employed to target the pathogen inhabiting the human system. Zur is one such prokaryotic transcriptional regulator whose function typically is to maintain Zn homeostasis but otherwise it does have certain moonlighting functions that make it an interesting protein to study. The point of the review is to know the structure and molecular mechanisms of Zur and to enlist the bacterial species that possess this protein, along with those that have it but are still waiting to be investigated. Additionally, we have touched upon the instances wherein this protein assists and helps evade many pathogens from the wrath of host-mediated nutritional immunity.

The motive was to look for the various roles that Zur plays, specifically in the pathogens. We have tried to touch upon as many pathogens as possible and have highlighted the crucial role that Zur plays for them. We would want to draw attention to those pathogens, in which the potential of this protein await investigation as far as its moonlighting functions are concerned. While concluding, we also hypothesise that this protein is not merely a Zn-dependent transcriptional regulator, rather, it is a master protein that is key for survival in many pathogens, and that it could be a potential antimicrobial target, but this fact certainly merits further investigation. 

## Data Availability

Data sharing not applicable.

## Abbreviations

mM: millimolar; fM: femtomolar; µM: micromolar; pM: picomolar.
